# Gray death: a powerful opioid combination leading to rapid fatality – correspondence

**DOI:** 10.1097/MS9.0000000000000310

**Published:** 2023-03-14

**Authors:** Tanvir Hasan, Saad A. Sami, Jaytirmoy Barmon, Mohammed K. Hossain, Talha B. Emran

**Affiliations:** a Department of Pharmacy, Faculty of Biological Sciences, University of Chittagong, Chittagong; b Department of Pharmacy, Faculty of Pharmacy, Varendra University, Rajshahi; c BCSIR Rajshahi Laboratories, Bangladesh Council of Scientific and Industrial Research, Rajshahi; d Department of Pharmacy, BGC Trust University Bangladesh, Chittagong, Bangladesh; e Department of Pharmacy, Faculty of Allied Health Sciences, Daffodil International University, Dhaka, Bangladesh

Gray death is a street name or a slang term that is frequently used to describe a mixture of illegal drugs – mainly synthetic opioids and other synthetic narcotics. Psychoactive components such as heroin, fentanyl, or U-47700 (an extremely strong synthetic opioid painkiller) are commonly found in drug cocktails. Occasionally, it is in combination with other substances like cocaine, amphetamines, or other synthetic designer drugs. This mixture resembles concrete powder or tiny rocks in appearance. It can be injected intravenously, processed into a fine powder, snorted intranasally, smoked, or consumed orally in tablet form^1^.

This illicit drug first appeared in the South at the beginning of 2017, mostly in Alabama and Georgia. This drug also spread in Ohio and Pennsylvania. The American citizens were the test subjects for this ‘mad science’^2^. Twenty persons in Argentina died in February 2022 from cocaine use linked with carfentanil^3^. From January 2017 to April 2017, the substance has been involved in at least 50 incidents and 17 overdoses in Georgia alone. In the county south of Columbus in 2021, Ohio State Troopers seized narcotics worth around $30 000, including quantities of the synthetic opioid combination^4^. The exact contents of ‘gray death’ may differ from batch to batch, making it even more deadly. The National Institute on Drug Abuse claims that it is not one drug but rather a mixture of several very strong opioids, including any that a drug dealer may have on hand. It generally includes the extremely potent addictive opioids Heroin, made from morphine found in the opium poppy (*Papaver somniferum*) plant. It also contains fentanyl, a synthetic opioid medicine up to 100 times stronger than other opioids, and U-47700. Therefore, a dangerous designer drug is made; its low dosages can be fatal or deadly. It may include a dangerous combination of other highly strong opioids and toxins, such as carfentanil which is 100 times stronger compared to fentanyl and 10 000 times stronger compared to morphine^5^.

The adverse effects of gray death can be mild to severe because no two batches are exactly the same in composition. Confusion, difficulty moving, tiredness, tremors, balance loss, seizures, mental fog, vomiting and nausea, myosis, spasms or generalized convulsion, and hypoventilation are some of the major side effects of gray death. Symptoms of gray death overdose include a faded or sweaty face, a languid body, grunting noises, purplish or bluish lips, shallow breathing, irregular pulse, and speech difficulties^4^. Multiple doses of the opioid-reversing drug naloxone (Narcan) may be needed to treat gray death overdose. It is frequently used to combat respiratory problems brought on by opioid overdose. Some patients may require a maximum of 10 doses for healing^6^. When using buprenorphine and naloxone to treat overdoses of strong narcotics from the fentanyl group, problems may frequently develop. The action of naloxone is hindered by opioids’ higher affinity for the µ-opioid receptor^5^.

When it comes to the potential of overdosing, gray death may be the scariest of all drugs. Nowadays, the National Institutes of Health, along with private organizations, are collaborating together on three distinct sectors: the development of enhanced overdose-reversing medications and preventative strategies to reduce death rates, the protection of lives and recovery through innovative pharmaceutical and technological remedies, and the discovery of effective, nonaddictive, and safe treatments for chronic pain^7^.

## Ethical approval

This case report has been reported in line with the CARE (CAse REport) guidelines.

## Patient consent

NA.

## Source of funding

None.

## Author contribution

T.H. and S.A.S.: conceptualization, data curation, writing – original draft preparation, reviewing, and editing; J.B.: data curation, writing – original draft preparation, reviewing, and editing; M.K.H.: visualization, writing – original draft preparation, reviewing, and editing; T.B.E.: conceptualization, writing – reviewing and editing, and visualization.

## Conflicts of interest disclosure

None.

## Research registration unique identifying number (UIN)

1. Name of the registry: not applicable.
2. Unique identifying number or registration ID: not applicable.
3. Hyperlink to your specific registration (must be publicly accessible and will be checked): not applicable.

## Guarantor

Talha Bin Emran, PhD, Associate Professor, Department of Pharmacy, BGC Trust University Bangladesh, Chittagong 4381,Bangladesh. Tel: +880 303 356 193, Fax: +880 312 550 224. https://orcid.org/0000-0003-3188-2272

## Data availability statement

NA.

## Provenance and peer review

Not commissioned, externally peer-reviewed.

## Declaration of competing interest

We have read and understood the policy on the declaration of interests and have no relevant interests to declare. The responsibility for the content lies with the author, and the views stated herein should not be taken to represent those of any organizations or groups with and for which he works.
